# Meningomylocele: An update

**DOI:** 10.4103/0970-1591.32072

**Published:** 2007

**Authors:** R. Kapoor, S. Agrawal

**Affiliations:** Department of Urology, Sanjay Gandhi Post Graduate Institute of Medical Science, Lucknow - 226 014, UP, India; * Department of Renal Transplantation, Sanjay Gandhi Post Graduate Institute of Medical Science, Lucknow - 226 014, UP, India

**Keywords:** Children, meningomyelocele, urodynamics

## Abstract

Therapy-resistant overactivity of detrusor or small capacity and poor compliance, will usually need to be treated by bladder augmentation. Ileal or colonic patches are used frequently for augmenting the bladder, with either intestinal segment appearing to be equally useful. Stomach is rarely used because of the associated complications, but it is the only available intestinal segment for patients with impaired renal function. Concerns regarding long-term effects of associated metabolic acidosis, including abnormalities in linear growth and bone metabolism are misplaced. Ureterocystoplasty offers an attractive urothelium-preserving alternative, avoiding the metabolic complications, mucus production and cancer risk of heterotopic epithelium associated with enterocystoplasty. Though ideal for patients with dilated ureter and nonfunctioning kidney, in patients with functioning kidney it carries added risks associated with transuretero-ureterostomy, mainly obstruction. Ureteral dilatation in meningomyelocele patients is avoidable with proper follow-up and treatment. Therefore they rarely should be candidates for this operation. Alternative urothelium-preserving techniques, such as auto augmentation and seromuscular cystoplasty, have not proven to be as successful as standard augmentation with intestinal segment. Work is in progress on various bioengineering techniques to culture and combine bladder cells in tissue culture for regeneration. Early efforts are exciting, but preliminary.

Meningomyelocele (MMC) affects as many as 1 in 1000 births, comprising the most common of pediatric neurogenic bladder and paralysis. Neurogenic bladder in these children presents with various patterns of detrusor-sphincter dysfunction within a wide range of severity. About 15% of neonates with myelodysplasia have no signs of neurological dysfunction at birth. However, there is a high chance of progressive changes in the dynamics of neurological lesions with time. Even babies with normal neuro-urological function at birth have a one in three risk of developing either detrusor sphincter dysynergia or denervation by the time they reach puberty. At birth, the majority of patients have normal upper urinary tract, but nearly 60% of them develop upper tract deterioration due to infection, bladder changes and reflux.[1–4] Morbidity in these children usually arises from the Chiari malformation, urological complications and orthopedic disabilities. Accordingly, children born with MMC preferably are evaluated and managed by an interdisciplinary team including pediatric urologist, neurosurgeon orthopedician and developmental pediatrician, as well as specialized nurses, physiotherapists, dietician and social workers. Together these professionals must interact with the patient and family from the time of diagnosis throughout childhood to develop plans for the management of medical and social issues arising from these conditions. Despite great advances in the care for patients with MMC, there remains a need for evidence-based outcome studies to define optimal management.

## DEFINITION

The term myelodysplasia includes a group of developmental anomalies that result from defects in neural tube closure. Lesions may include spina bifida occulta, meningocele, meningomyelocele or lipo-meningomyelocele. MMC is by far the most common defect seen and the most detrimental. The term MMC refers to incomplete closure of the vertebral column during embryogenesis, resulting in exposure of the meninges and spinal cord. It is always associated with a constellation of findings - Chiari malformation Type II, which includes hindbrain herniation into the upper cervical canal, dysgenesis or agenesis of corpus callosum, neuronal migration of varying degree and hydrocephalus.[5] Additionally, different growth rates between the vertebral bodies and the elongating spinal cord can introduce a dynamic factor to the lesion. Scar tissue surrounding the cord at the site of meningocele closure can tether the cord during the gradual process of growth, producing clinical manifestations of tethered cord syndrome at a later stage.

## GENETICS

MMC and other neural tube defects represent examples of multifactorial inheritance. Parents of an affected infant have a 1 in 30 chance of producing a second affected offspring. An affected parent, if able to father children has a 3-4% chance of having an affected child. The risk for inheriting this disorder among first degree relative is 1 in 200 and amongst second degree relative is 1 in 100. Maternal folic acid deficiency is an environmental factor strongly associated with neural tube defects (NTDs).[6] Supplementation with multivitamin containing 400 μg of folic acid prevents the occurrence of more than 50% of NTDs when taken before conception and continued throughout the first trimester of pregnancy.[7] Daily consumption of 4000 μg (4 mg) of folic acid periconceptionally is recommended for prevention of NTDs in women who have had a previous pregnancy affected by NTD.[8]

## CLASSIFICATION

The purpose of any classification system is to facilitate the understanding and management of the underlying pathology. In children, the spinal level and extent of congenital lesion are poorly correlated with the clinical outcome. Urodynamic and functional classifications have therefore been more practical for defining the extent of the pathology and planning treatment in children.

Bladder and sphincter work in tandem to make a single functional unit. According to the nature of neurological deficit, the bladder and sphincter may exhibit increased or decreased activity:

1. Bladder is: a) overactive [uninhibited contractions] b) low capacity with poor compliance or c) Underactive/inactive with no effective contractions.
2. The outlet (urethra and sphincter) may be independently: a) overactive causing functional obstruction or b) paralytic with no resistance to urinary flow.

These conditions may present in different combinations. This is mainly a classification based on urodynamic findings. In meningomyelocele, most patients will present with overactive detrusor and detrusor sphincter dysynergia.

## ANTENATAL ISSUES

Antenatal diagnosis of MMC is made on elevated maternal alpha-protein levels or by sonographic evaluation of NTDs. Biochemical tests include maternal serum alpha fetoprotein (MSAFP), amniotic fluid alpha fetoprotein and amniotic fluid acetyl cholinesterase. The latter two are used as confirmatory tests and should not be regarded as part of routine screening of women at low risk of neural tube defects. Screening by elevated MSAFP (2-2.5 times the median value for gestational age) measured at 16-18 weeks of gestation can detect 56-91% of affected fetuses.[9] Ultrasound should be used both as a screening test and as a follow-up test after positive MSAFP screening. It has a sensitivity of 79-96% and specificity of 90-100%.[10]

While technically possible, surgery for MMC is presently not being performed via a fetoscopic approach.[11] Open fetal surgery requires careful planning and is fraught with high complication rate. In different studies, despite the early repair, patterns of persistent abnormal function have been elicited.[12,13]

## NEWBORN MANAGEMENT

For families interested in continuing the pregnancy, proper counseling including information about fetal repair, other maternal care issues and prognosis of children should be explained. There is evidence for a better motor level and ambulatory outcome following delivery by elective cesarean section in fetuses who have a bulging lumbosacral MMC and presence of motor function in the affected spinal cord segments in-utero. Fetuses with flat MMC or a large lesion with no motor function below T12, often do not benefit from elective cesarean section.[14]

After birth, the infant should be handled with nonlatex gloves as they are increasingly prone to latex allergy/sensitivity. Preterm newborns with MMC are more likely to have congenital heart disease and /or syndromic causes (e.g., trisomy 13 or 18) than term newborns.[15] Complete neurological examination should be obtained and early operative repair (within 48h) should be considered for prevention of infection. There should be a ready access to urological, neurosurgical, orthopedic and ICU care facilities.

## INITIAL UROLOGICAL ASSESSMENT

All these infants must be assumed to have a neurogenic bladder because only 5% of MMC can void spontaneously by typical age of toilet training.[16] However, specific abnormalities vary considerably and are not predicted by the level of spinal cord defect or the neurological examination. Furthermore, bladder dynamics are temporarily affected by spinal cord surgery, which often results in areflexia with urinary retention. Many patients initially require intermittent catheterization which could be performed at sufficiently frequent intervals to avoid bladder over-distension. Spontaneous voiding may resume within a few days. Baseline urological investigations include renal/bladder sonography and a micturating cystourethrogram (MCU) which may already demonstrate evidence of neurogenic bladder dysfunction. Hydronephrosis is found in 7-30% of infants[17,18] although sometimes this upper tract dilatation only reflects temporary urinary retention. Reflux is observed in 20% of infants.[17] Another consideration in newborn males is circumcision. Because most children eventually require intermittent catheterization, there may be an advantage of circumcision facilitating access to the meatus.

### Urodynamic evaluation

In 1981, McGuire *et al*,[19] observed that upper tract changes occurred when intravesical pressure (DLPP) during filling or voiding exceeded 40 cm water. They further noted that the cause of elevated pressure was fixed outlet resistance from denervation of external sphincter, resulting in bladder outlet obstruction. This explained the observations on serial imaging that an initially smooth-walled bladder could become trabeculated and assume vertically oriented “Christmas tree” configuration. Based on these findings, patients with MMC can be classified into high- and low-risk groups according to their DLPP. Boston group reported that newborn UDS were predictive of patient outcomes. Eighty per cent of infants believed to be at high risk experienced upper tract changes when followed up expectantly.[20]

Serial radiological imaging for detection of secondary evidence of high detrusor pressures is an effective alternative to urodynamics-based management. In more than 50% patients, observed with periodic radiological imaging, febrile urinary tract infection, hydronephrosis or reflux can be observed by three years of age.[18] However, appropriate intervention when such problems/changes occur has resulted in deterioration in only 1-5% of renal units.[18] Thus underscoring the importance of these studies. Use of nuclear scintigraphy in urodynamics or radiological-based management protocols has not been reported till date.

Urodynamics may be criticized for lack of standards of performance and interpretation of tests which may lead to unnecessary interventions. McGuire *et al*,[19] stressed the importance of DLPP more than 40 cm of water as determined by “slow fill” cystometry. Joseph[21] emphasized the importance of infusion rate in DLPP determination. He found that fill rate of 20% of estimated bladder capacity per minute, was more often associated with a DLPP more than 40 cm of water, when compared to fill rate of 2% of estimated bladder capacity in the same patients. Decter and Harpster[22] found that DLPP was also influenced by catheter size and gravity drip versus infusion pump filling. Furthermore, the urodynamic patterns can change over time, with conversion of a low-risk DLPP to one greater than 40 cm H2O, raising questions regarding the best timing for initial and follow-up studies. Despite these controversies, many centers manage infants with newborn testing with urodynamic evaluation and preemptive intervention. Until controlled studies comparing management strategies are available, well done urodynamic studies should help to identify most patients at risk and preemptive therapy in these cases may prevent many adverse effects from neurological deficit.

### Newborn urodynamics

Initial urodynamic testing is performed soon after the state of spinal shock subsequent to neurological closure resolves, usually around six weeks of age. It is reasonable to infuse contrast medium or saline via a pump with a fill rate based on 10% of estimated bladder capacity (approx 5 ml/min). Urodynamic evaluation with electromyographic (EMG) and associated videoscopic evaluation are ideal. Tests performed in an agitated infant are difficult to interpret and several fillings may be needed to verify the findings. A review of the literature reveals that there are few studies that have attempted to characterize urodynamic findings in newborns with MM, 12-32% of patients had normal studies. Detrusor overactivity was noted in 40-76%, the rest revealed underactivity/areflexic detrusor with preserved or diminished compliance. External sphincter had normal EMG activity in approximately 40% and the rest showed evidence of partial or complete denervation.[23–25]

### Changing urodynamic pattern in MMC

Spindel *et al*,[26] first reported neuro-urological changes in children detected by serial urodynamic testing, which occurred in 37% of patients during the first three years of life and usually within the first 12 months. Specifically, they evaluated external sphincteric activity and found evidence of new or progressive denervation in half of the cases, whereas the rest 50% showed improved innervation. Patients with deterioration had radiological findings of a tethered cord. Subsequent study at the same institute reported that neurosurgical intervention resulted in improvement or stabilization of urodynamic findings in 90% of children in whom it was performed.[27] Overall, the risk of deterioration leading to consideration for additional neurosurgical procedure was 10% during the first year of life. Among infants initially found to have normal external sphincteric innervation, partial or complete denervation developed in 27%. At the same time, 46% of patients with detrusor sphincter synergia at birth later had dysynergia.

Other researchers have focused attention on detrusor responses. Roach *et al*,[28] observed that 32% of infants with an initially low DLPP and normal upper tracts converted to a high DLPP within six months. Sillen *et al*,[25] performing urodynamic testing at 1, 4 and 10 months, found that 40% of patients experienced changes typically with worsening compliance, which occurred most often within one and four months. The risk of neuro-urological deterioration is best exemplified by a study of 25 newborns with normal urodynamics.[23] Initial assessment was done at 14 days of age when neurological evaluation suggested resolution of spinal shock. Follow-up UDS were performed annually. Deterioration occurred in 40% of cases, all within six years of age. In most cases UDS detected these changes before radiological or neurological changes. Subsequent spinal cord detethering restored normal function in 25%. The patients who remained stable during follow-up were ambulatory, voided spontaneously and achieved satisfactory urinary and fecal continence. It is reasonable to conclude that the neuro-urological status changes in some patients, often within the first 12 months of life. Of greatest concern is loss of external sphincteric innervation, leading to fixed elevated outlet resistance. Observation that neurosurgical intervention restores function in some cases potentially increases the responsibility of the urologist to detect changes on UDS, before these changes precede other clinical signs of cord tethering. Optimal follow-up schedule and appropriate time for detethering to restore changing detrusor-sphincteric functions, still remains unclear. Longitudinal, controlled multi-institutional studies are needed to define these parameters.

## TREATMENT

From the outset, the goals of urological management are:

1. To minimize damage to the upper tracts and bladder.
2. To minimize the social disabilities, from the consequences of neurological deficit and neurogenic bladder dysfunction.

Management of children with MMC with a neurogenic bladder requires constant observation and adaptation to new problems.

### Anticholinergics and intermittent catheterization

High-risk infants, that is, those with DLPP greater than 40 cm of water or DSD, are started on anticholinergics (a regime of 0.2 mg/kg dose of oxybutynin twice daily) and intermittent catheterization every three hours throughout the day. In many cases, catheterization at least once during the night (as the infant awakens for feeding) is also recommended. Advocates justify this approach to minimize the deleterious effects of high intravesical pressure, noting that in more than 50% of patients who are observed with periodic radiological imaging, febrile urinary tract infection (UTI), hydronephrosis or reflux develops by three years of age.[18] Eighty per cent of infants believed to be at high risk experienced upper tract changes when followed up expectantly.[20] Recent studies have suggested that early intervention may protect both upper tracts and bladder, diminishing the eventual need for augmentation. Kafer *et al*,[29] compared high-risk patients followed expectantly with a subsequent group undergoing early intervention and found that augmentation rate decreased from 41% to 17% in the intervention group. Wu *et al*,[17] similarly noted fewer augmentations when children began therapy during the first year of life. Though no study reported so far has investigated patient and family acceptance of intermittent catheterization based on the age at which it is instituted, it is generally believed that early initiation of intermittent catheterization increases patient and family understanding and acceptance.[30] Urodynamic findings are repeated six weeks later to verify a sufficient anticholinergic response and to compare routine volumes obtained during catheterization at home with safe storage volumes determined by testing. It is prudent to monitor these children with renal sonography every six months and MCU annually for the next several years. Urodynamics are repeated if new hydronephrosis, reflux or unexplained febrile UTIs develop.[16] Infants with a low DLPP on initial urodynamics most often are left on diaper drainage without intermittent catheterization. These patients are also followed up with renal sonography and MCU at age six months with sonograms to be repeated annually. The UDS are repeated in those whom upper tract deterioration or febrile UTIs develop.

### Vesicostomy

Widely practiced in the past, it is another option for early management of high intravesical pressures. Indications include intolerance to anticholinergic medications or the failure of pharmacotherapy to improve detrusor compliance sufficiently and maintain low storage pressure despite intermittent catheterization. Rarely, surgery is performed because the parents are unable to perform catheterization owing to their work schedules or other social considerations.

### Botulinum toxin injections

Useful in neurogenic bladders which are refractory to anticholinergics and remain in a small-capacity, high-pressure state. Treatment seems to be more effective on bladders with a more active component.[31,32] However, there is lack of prospective controlled trials regarding its clinical effect and usage.

### Urinary tract infection

Urinary tract infections are common in children with neurogenic bladders. Bacteriuria is found in 60-80% of children with MMC, especially in those undergoing intermittent catheterization and typically does not require treatment.[33,34] Symptomatic UTIs should be treated. Recurrent febrile UTI has been estimated to occur in approximately 20% of patients.[16]

Antibiotic prophylaxis is used in children who have upper tract changes of reflux or hydronephrosis.[35]

### Bladder augmentation

Therapy-resistant overactivity of detrusor or small capacity and poor compliance, will usually need to be treated by bladder augmentation. Ileal or colonic patches are used frequently for augmenting the bladder, with either intestinal segment appearing to be equally useful. Stomach is rarely used because of the associated complications, but it is the only available intestinal segment for patients with impaired renal function.[36] Concerns regarding long-term effects of associated metabolic acidosis, including abnormalities in linear growth and bone metabolism are misplaced.[37] Ureterocystoplasty offers an attractive urothelium-preserving alternative, avoiding the metabolic complications, mucus production and cancer risk of heterotopic epithelium associated with enterocystoplasty. Though ideal for patients with dilated ureter and nonfunctioning kidney, in patients with functioning kidney it carries added risks associated with transuretero-ureterostomy, mainly obstruction. Ureteral dilatation in MMC patients is avoidable with proper follow-up and treatment. Therefore, they rarely should be candidates for this operation. Alternative urothelium-preserving techniques, such as auto-augmentation and seromuscular cystoplasty, have not proven to be as successful as standard augmentation with intestinal segment.[38] Work is in progress on various bioengineering techniques to culture and combine bladder cells in tissue culture for regeneration. Early efforts are exciting, but preliminary.

### Bladder outlet procedures

Children with detrusor overactivity but with underactive sphincters will be severely incontinent. Initial treatment is intermittent catheterization with anticholinergics drugs (as it may reduce the degree of incontinence and offers a much better control over UTIs). At a later stage, the outlet resistance will be increased in order to render them continent. Medical treatment like alpha receptor stimulation has not proven to be effective.[39] Surgical measures including bladder neck reconstruction or urethral reconstruction may be required along with bladder augmentation. But these measures may complicate transurethral catheterization so augmentation with surgical closure of the bladder neck may be required primarily or as a secondary procedure. In this situation, a continent stoma will be required. An abdominal wall continent stoma may be particularly beneficial to the wheelchair-bound MMC patient, who may often have difficulty with urethral catheterization or who is dependent on others for intermittent catheterization.

### Tethered cord syndrome

Patients with MMC and tethered cord syndrome of delayed onset usually present with bladder spasticity and orthopedic foot deformities. It is prudent to consider untethering of cord at the time of onset of symptoms to prevent further deterioration.[40]

## CONCLUSION

**Figure 1:**
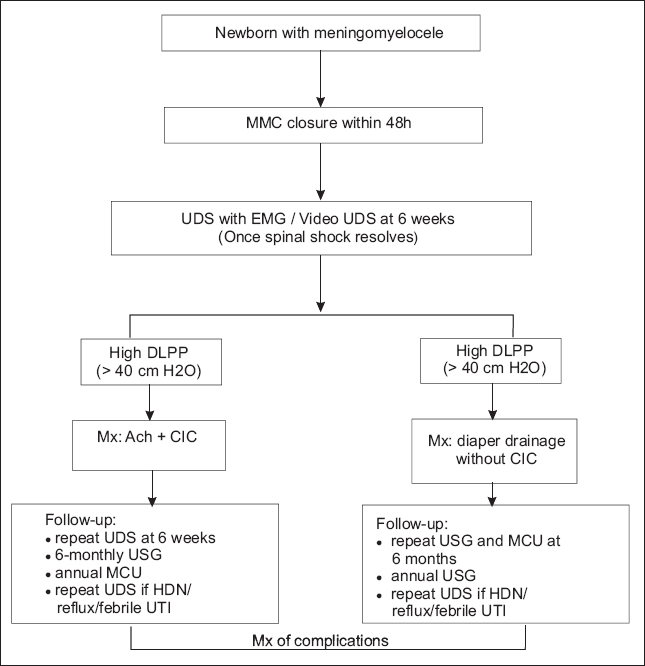
Management algorithm of patients with meningomyelocele

Newborns with MMC pose a management challenge as few are born with and maintain normal bladder and sphincter function. Interdisciplinary teams of neurosurgeons, urologists, pediatricians orthopedicians, ICU staff, paramedical and nursing staff along with rehabilitation workers are required for optimal management of problems associated with such children. Urodynamics and radiological imaging play an essential role in ascertaining the nature of urological involvement in such patients. Worldwide, management is tailored to these findings. Regardless of whether management is primarily based on urodynamics (with preemptive intervention), radiological imaging (with intervention for development of adverse changes) or a combination of the two, all children with MM require close surveillance for urologic problems, especially during the first few years of life. Goals to protect the bladder and upper tracts and eventually to attain continence, ideally by school age, are discussed with the family from the outset and reinforced during periodic follow-up visits. In the absence of established standardized management protocols, we propose a management algorithm of such patients based on our own institutional experience and literature-based evidence [Figure 1].

## References

[1] Bauer SB, Whitaker RH, Woodard JR (1985). The management of spina bifida from birth onwards. Paediatric urology.

[2] Bauer SB, King LR (1988). Early evaluation and management of children with spina bifida. Urologic surgery in neonates and young infants.

[3] Wilcock AR, Emery JL (1970). Deformities of the renal tract in children with meningomyelocele and hydrocephalus, compared with those of children showing no such deformities. Br J Urol.

[4] Hunt GM, Whitaker RH (1987). The pattern of congenital renal anomalies associated with neural-tube defects. Dev Med Child Neurol.

[5] McLone DG, Knepper PA (1989). The cause of Chiari II malformation: A unified theory. Pediatr Neurosci.

[6] (1999). Folic acid for the prevention of neural tube defects. American Academy of Pediatrics Committee on Genetics. Pediatrics.

[7] (1991). Centers for Disease Control and Prevention. Use of folic acid for prevention of spina bifida and other neural tube defects: 1983-1991. MMWR Morb Mortal Wkly Rep.

[8] (1992). Centers for Disease Control and Prevention. Recommendations for the use of folic acid to reduce the number of cases of spina bifida and other neural tube defects. MMWR Morb Mortal Wkly Rep.

[9] Milunsky A, Alpert E (1984). Results and benefits of a maternal serum alpha-fetoprotein screening program. JAMA.

[10] Tyrrell S, Howel D, Bark M, Allibone E, Lilford RJ (1988). Should maternal alpha-fetoprotein estimation be carried out in centers where ultrasound screening is routine? A sensitivity analysis approach. Am J Obstet Gynecol.

[11] Coplen DE (1997). Prenatal intervention for hydronephrosis. J Urol.

[12] Holmes N, Harrison MR, Baskin LS (2001). Fetal surgery for posterior urethral valves: Long-term postnatal outcomes. Pediatrics.

[13] Harrison MR, Golbus MS, Filly RA, Anderson RL, Flake AW, Rosen M (1987). Fetal hydronephrosis: Selection and surgical repair. J Pediatr Surg.

[14] Luthy DA, Wardinsky T, Shurtleff DB, Hollenbach KA, Hickok DE, Nyberg DA (1991). Cesarean section before the onset of labor and subsequent motor function in infants with meningomyelocele diagnosed antenatally. N Engl J Med.

[15] Charney EB, Weller SC, Sutton LN, Bruce DA, Schut LB (1985). Management of the newborn with myelomeningocele: Time for a decision making process. Pediatrics.

[16] Snodgrass TW, Adams R (2004). Initial urologic management of myelomeningocele. Urol Clin North Am.

[17] Wu HY, Baskin LS, Kogan BA (1997). Neurogenic bladder dysfunction due to myelomeningocele: Neonatal versus childhood treatment. J Urol.

[18] Hopps CV, Kropp KA (2003). Preservation of renal function in children with myelomeningocele managed with basic newborn evaluation and close follow-up. J Urol.

[19] McGuire EJ, Woodside JR, Borden TA, Weiss RM (1981). Prognostic value of urodynamic testing in myelodysplastic patients. J Urol.

[20] Edelstein RA, Bauer SB, Kelly MD, Darbey MM, Peters CA, Atala A (1995). The long-term urologic response of neonates with myelodysplasia treated proactively with intermittent catheterization and anticholinergic therapy. J Urol.

[21] Joseph DB (1992). The effect of medium-fill and slow-fill saline cystometry on detrusor pressure in infants and children with myelodysplasia. J Urol.

[22] Decter DM, Harpster L (1992). Pitfalls in determination of leak point pressure. J Urol.

[23] Tarcan T, Bauer S, Olmedo E, Khoshbin S, Kelly M, Darbey M (2001). Long-term followup of newborns with myelodysplasia and normal urodynamic findings: Is followup necessary?. J Urol.

[24] Sidi AA, Dykstra DO, Gonzalez R (1986). The value of urodynamic testing in the management of neonates with myelodysplasia: A prospective study. J Urol.

[25] Sillen U, Hansson E, Hermansson G, Hjalmas K, Jacobsson B, Jodal U (1996). Development of the urodynamic pattern in infants with myelomeningocele. BJU Int.

[26] Spindel MR, Bauer SB, Dyro FM, Krarup C, Khoshbin S, Winston KR (1987). The changing neurourologic lesion in myelodysplasia. JAMA.

[27] Lais A, Kasabian NG, Dyro FM, Scott RM, Kelly MD, Bauer SB (1993). The neurosurgical implications of continuous neurourological surveillance of children with myelodysplasia. J Urol.

[28] Roach MB, Switters DM, Stone AR (1993). The changing urodynamic pattern in infants with myelomeningocele. J Urol.

[29] Kaefer M, Pabby A, Kelly M, Darbey M, Bauer SB (1999). Improved bladder function after prophylactic treatment of the high risk neurogenic bladder in newborns with myelomeningocele. J Urol.

[30] McGuire EJ, Woodside JR, Borden TA, Weiss RM (2002). Prognostic value of urodynamic testing in myelodysplastic patients. 1981. J Urol.

[31] Smith CP, Somogyi GT, Chancellor MB (2002). Emerging role of botulinum toxin in the treatment of neurogenic and non-neurogenic voiding dysfunction. Curr Urol Rep.

[32] Leippold T, Reitz A, Schurch B (2003). Botulinum toxin as a new therapy option for voiding disorders: Current state of the art. Eur Urol.

[33] Hansson S, Caugant D, Jodal U, Svanborg-Eden C (1989). Untreated asymptomatic bacteriuria in girls: I-Stability of urinary isolates. BMJ.

[34] Hansson S, Jodal U, Lincoln K, Svanborg-Eden C (1989). Untreated asymptomatic bacteriuria in girls: II - Effect of phenoxymethylpenicillin and erythromycin given for intercurrent infections. BMJ.

[35] Schlager TA, Anderson S, Trudell J, Hendley JO (1998). Nitrofurantoin prophylaxis for bacteriuria and urinary tract infection in children with neurogenic bladder on intermittent catheterization. J Pediatr.

[36] Nguyen DH, Mitchell ME (1991). Gastric bladder reconstruction. Urol Clin North Am.

[37] Mingin G, Maroni P, Gerharz WE, Woodhouse CR, Baskin LS (2004). Linear growth after enterocystoplasty in children and adolescents: A review. World J Urol.

[38] Duel BP, Gonzalez R, Barthold JS (1998). Alternative techniques for augmentation cystoplasty. J Urol.

[39] Naglo AS (1982). Continence training of children with neurogenic bladder and detrusor hyperactivity: Effect of atropine. Scan J Urol Nephrol.

[40] Phuong LK, Schoeberi KA, Raffel C (2002). Natural history of tethred cord in patients with meningomyelocele. Neurosurgery.

